# Detection of a pair density wave state in UTe_2_

**DOI:** 10.1038/s41586-023-05919-7

**Published:** 2023-06-28

**Authors:** Qiangqiang Gu, Joseph P. Carroll, Shuqiu Wang, Sheng Ran, Christopher Broyles, Hasan Siddiquee, Nicholas P. Butch, Shanta R. Saha, Johnpierre Paglione, J. C. Séamus Davis, Xiaolong Liu

**Affiliations:** 1 LASSP, Department of Physics, Cornell University, Ithaca, NY USA; 2 Department of Physics, University College Cork, Cork, Ireland; 3 Clarendon Laboratory, University of Oxford, Oxford, UK; 4 Department of Physics, Washington University in St. Louis, St. Louis, MO USA; 5 Maryland Quantum Materials Center, University of Maryland, College Park, MD USA; 6 NIST Center for Neutron Research, Gaithersburg, MD USA; 7 Canadian Institute for Advanced Research, Toronto, Ontario Canada; 8 Max Planck Institute for Chemical Physics of Solids, Dresden, Germany; 9 Department of Physics and Astronomy, University of Notre Dame, Notre Dame, IN USA; 10 Stavropoulos Center for Complex Quantum Matter, University of Notre Dame, Notre Dame, IN USA

**Keywords:** Superconducting properties and materials, Electronic properties and materials, Topological matter

## Abstract

Spin-triplet topological superconductors should exhibit many unprecedented electronic properties, including fractionalized electronic states relevant to quantum information processing. Although UTe_2_ may embody such bulk topological superconductivity^1–11^, its superconductive order parameter Δ(**k**) remains unknown^12^. Many diverse forms for Δ(**k**) are physically possible^12^ in such heavy fermion materials^13^. Moreover, intertwined^14,15^ density waves of spin (SDW), charge (CDW) and pair (PDW) may interpose, with the latter exhibiting spatially modulating^14,15^ superconductive order parameter Δ(**r**), electron-pair density^16–19^ and pairing energy gap^17,20–23^. Hence, the newly discovered CDW state^24^ in UTe_2_ motivates the prospect that a PDW state may exist in this material^24,25^. To search for it, we visualize the pairing energy gap with μeV-scale energy resolution using superconductive scanning tunnelling microscopy (STM) tips^26–31^. We detect three PDWs, each with peak-to-peak gap modulations of around 10 μeV and at incommensurate wavevectors **P**_*i*=1,2,3_ that are indistinguishable from the wavevectors **Q**_*i*=1,2,3_ of the prevenient^24^ CDW. Concurrent visualization of the UTe_2_ superconductive PDWs and the non-superconductive CDWs shows that every **P**_*i*_:**Q**_*i*_ pair exhibits a relative spatial phase *δϕ* ≈ π. From these observations, and given UTe_2_ as a spin-triplet superconductor^12^, this PDW state should be a spin-triplet PDW^24,25^. Although such states do exist^32^ in superfluid ^3^He, for superconductors, they are unprecedented.

## Abstract (web summary)

A spin-triplet pair density wave is discovered in the candidate topological superconductor UTe_2_ using superconductive scanning tunnelling microscopy tips.

## Main

**Fig. 1:**
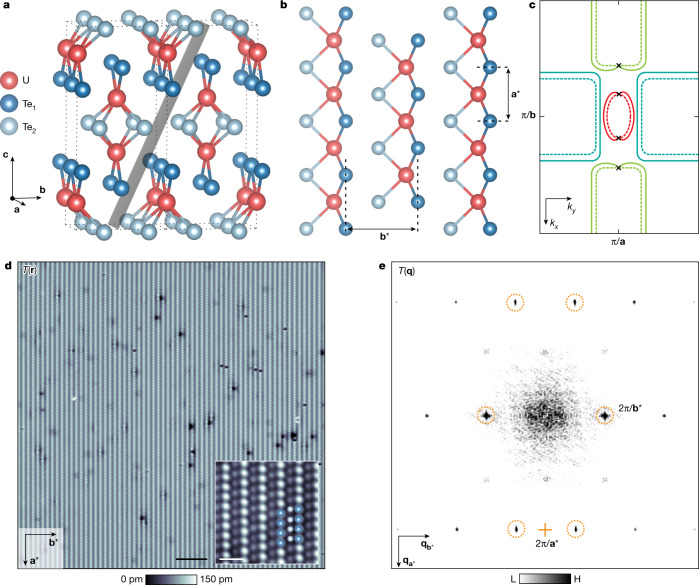
Momentum-space and real-space characteristics of UTe_2_. **a**, Schematic crystal-lattice structure of UTe_2_ oriented to the primary unit cell vectors **a**,**b** and **c**. The (0–11) cleave plane of UTe_2_ is indicated schematically by the grey-shaded plane. **b**, Schematic of elemental identities and atomic sites and unit cell of the (0–11) termination layer of cleaved UTe_2_. **c**, Schematic Fermi surface in the (*k*_*x*_, *k*_*y*_) plane at *k*_*z*_ = 0 for UTe_2_ is indicated by dashed curves. A schematic example of one possible superconductive order parameter magnitude is indicated by solid curves representing the magnitude of energy gap Δ(**k**). Here, for pedagogic purposes only, is presented a chiral, spin-triplet superconductor with energy gap nodes along the **a** axis or *k*_*x*_ axis. **d**, Typical topographic image *T*(**r**) of UTe_2_ (0–11) surface measured with a superconducting tip at *T* = 280 mK (*I*_s_ = 0.5 nA, *V*_s_ = 30 mV). Inset, measured high-resolution *T*(**r**) at low junction resistance (*I*_s_ = 3 nA, *V*_s_ = 5 mV), clarifying two types of Te atom. Scale bars, 5 nm (main), 1 nm (inset). **e**, Measured *T*(**q**), the Fourier transform of *T*(**r**) in **d**, with the surface reciprocal-lattice points labelled as dashed orange circles, which are consistent with simulated results (Extended Data Fig. 2).

Bulk Cooper-pair condensates are definitely topological when their superconductive or superfluid order parameters exhibit odd parity^33,34^ Δ(**k**) = −Δ(−**k**) with spin-triplet pairing. This situation is epitomized by liquid ^3^He, the only known bulk topological Cooper-pair condensate^35,36^. Although no bulk superconductor exhibits an unambiguously topological Δ(**k**), attention has recently focused on the compound UTe_2_ as a promising candidate^1–12^. This material is superconducting below the critical temperature *T*_c_ = 1.65 K. Its extremely high critical magnetic field and the minimal suppression of the Knight shift^3^ on entering the superconductive state both imply spin-triplet superconductivity^1,2^. Temperature^4^, magnetic field^4,5^ and angular dependence^5^ of the superconductive quasiparticle thermal conductivity are all indicative of a superconducting energy gap with point nodes^4–6^. In the superconductive phase, evidence for time-reversal symmetry breaking is provided by polar Kerr rotation measurements^7^ but is absent in muon-spin-rotation studies^8^. Furthermore, the superconductive electronic structure when visualized at opposite mesa edges at the UTe_2_ (0–11) surface breaks chiral symmetry^9^. Dynamically, UTe_2_ seems to contain both strong ferromagnetic and antiferromagnetic spin fluctuations^10,11^ relevant to superconductivity. Together, these results are consistent with a spin-triplet and, thus, odd-parity, nodal, time-reversal symmetry breaking, chiral superconductor^12^. Figure 1a shows a schematic of the crystal structure of this material, whereas Fig. 1c is a schematic of the Fermi surface in the (*k*_*x*_, *k*_*y*_) plane at *k*_*z*_ *=* 0 (dashed lines; ref. ^37^). An exemplary order parameter Δ(**k**) proposed^5^ for UTe_2_ is also shown schematically in Fig. 1c (solid lines), but numerous others have been proposed^12^, including that of a PDW state^24,25^. In theory, this PDW, if generated by time-reversal and surface-reflection symmetry breaking, is a spin-triplet PDW^25^. Such a state is unknown for superconductors but occurs in topological superfluid ^3^He (ref. ^32^).

## PDW visualization

In general, a PDW state is a superconductor but with a spatially modulating superconductive order parameter^14,15^. Absent flowing currents or magnetic fields, a conventional spin-singlet superconductor has an order parameter

1 $${\Delta }_{{\rm{S}}}({\bf{r}})={\Delta }_{0}{{\rm{e}}}^{{\rm{i}}{\phi }_{{\rm{S}}}}$$

for which *ϕ*_S_ is the macroscopic quantum phase and Δ_0_ the amplitude of the many-body condensate wavefunction. A unidirectional PDW modulates such an order parameter at wavevector **P** as

2 $${\Delta }_{{\rm{P}}}\left({\bf{r}}\right)=\Delta \left({\bf{r}}\right){{\rm{e}}}^{{\rm{i}}{\bf{P}}\cdot {\bf{r}}}+{\Delta }^{* }\left({\bf{r}}\right){{\rm{e}}}^{-{\rm{i}}{\bf{P}}\cdot {\bf{r}}}$$

meaning that the electron-pairing potential varies spatially. By contrast, a unidirectional CDW modulates the charge density at wavevector **Q** such that

3 $${\rho }_{{\rm{Q}}}({\bf{r}})=\rho \left({\bf{r}}\right){{\rm{e}}}^{{\rm{i}}{\bf{Q}}\cdot {\bf{r}}}+{\rho }^{* }\left({\bf{r}}\right){{\rm{e}}}^{-{\rm{i}}{\bf{Q}}\cdot {\bf{r}}}$$

The simplest interactions between these three orders can be analysed using a Ginzburg–Landau–Wilson free-energy density functional

4 $${\mathscr{F}}=\lambda [\,{\rho }_{{\rm{Q}}}{\Delta }_{{\rm{S}}}^{\ast }{\Delta }_{{\rm{P}}}+{\rm{c}}\,.{\rm{c}}.]$$

representing the lowest-order coupling between superconductive and density wave states.

There are two elementary possibilities: (1) if Δ_S_(**r**) and Δ_P_(**r**) are the predominant orders, they generate charge modulations of forms ${\rho }_{{\rm{P}}}\left({\bf{r}}\right)\propto {\Delta }_{{\rm{S}}}^{* }{\Delta }_{{\rm{P}}}+{\Delta }_{-{\rm{P}}}^{* }{\Delta }_{{\rm{S}}}$ and ${\rho }_{2{\rm{P}}}\left({\bf{r}}\right)\propto {{\Delta }_{-{\rm{P}}}^{* }\Delta }_{{\rm{P}}}$, that is, two induced CDWs controlled by the wavevector of the PDW; (2) if Δ_S_(**r**) and *ρ*_Q_(**r**) are predominant orders, they generate modulations ${\Delta }_{{\rm{Q}}}\left({\bf{r}}\right)\propto {\Delta }_{{\rm{S}}}^{* }{\rho }_{{\rm{Q}}}$, that is, a PDW induced at the wavevector of the CDW. In either case, the PDW state described by equation (2) subsists.

To explore UTe_2_ for such physics, it is first necessary to simultaneously visualize any coexisting CDW and PDW states. Recent experimental advances have demonstrated two techniques for visualizing a PDW state. In the first^16–19^, the condensed electron-pair density at location **r**, *n*(**r**), can be visualized by measuring the tip-sample Josephson critical-current squared ${I}_{{\rm{J}}}^{2}\left({\bf{r}}\right)$, from which

5 $$n({\bf{r}})\propto {I}_{{\rm{J}}}^{2}\left({\bf{r}}\right){R}_{{\rm{N}}}^{2}({\bf{r}})$$

in which *R*_N_(**r**) is the normal-state junction resistance. In the second PDW visualization technique^17,20–23^, the magnitude of the energy gap in the sample, Δ(**r**), is defined as half the energy separation between the two superconductive coherence peaks in the density of electronic states *N*(*E*). These occur in tunnelling conductance at signed energies Δ_+_(**r**) and Δ_−_(**r**) such that

6 $$\Delta ({\bf{r}})\equiv [{\Delta }_{+}({\bf{r}})-{\Delta }_{-}({\bf{r}})]/2$$

This can be visualized using either normal-insulator-superconductor (NIS) tunnelling^20–22^ or superconductor-insulator-superconductor (SIS) tunnelling from a superconductive STM tip^17,23,29^ whose superconductive gap energy, Δ_tip_, is known a priori.

## CDW visualization in normal-state UTe_2_

**Fig. 2:**
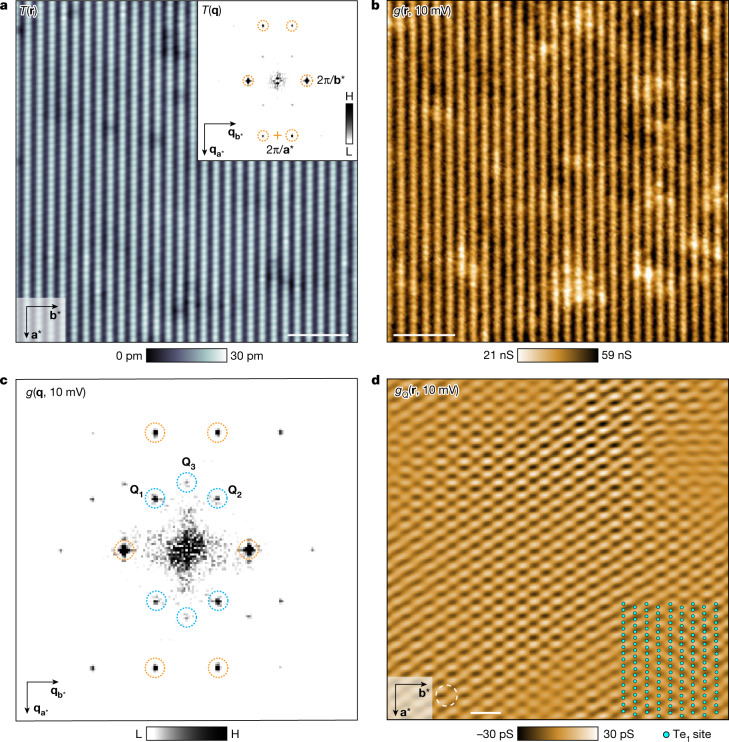
Visualizing the normal-state CDW of UTe_2_. **a**, Typical topographic image *T*(**r**) of (0–11) surface measured at 4.2 K with a non-superconducting STM tip (*I*_s_ = 1 nA, *V*_s_ = −30 mV). Inset, measured *T*(**q**), the Fourier transform of the topographic image obtained simultaneously as **b**. Reciprocal-lattice points labelled in dashed orange circles. Scale bar, 4 nm. **b**, Differential conductance image *g*(**r**, 10 mV) measured at 4.2 K. Scale bar, 4 nm. **c**, Fourier transform *g*(**q**, 10 mV) from *g*(**r**, 10 mV) in **b**. Three incommensurate CDW peaks at **Q**_1,2,3_ labelled by dashed blue circles. **d**, Measured density-of-states modulations *g*_Q_(**r**, 10 mV) only at the wavevectors **Q**_1,2,3_. This is a highly typical image of the incommensurate CDW state of UTe_2_ (Methods and Extended Data Fig. 1). Te_1_ atomic locations of UTe_2_ (0–11) surface shown as overlay. The filter size of the inverse Fourier transform is 14 Å. Scale bar, 2 nm.

**Extended Data Fig. 1:**
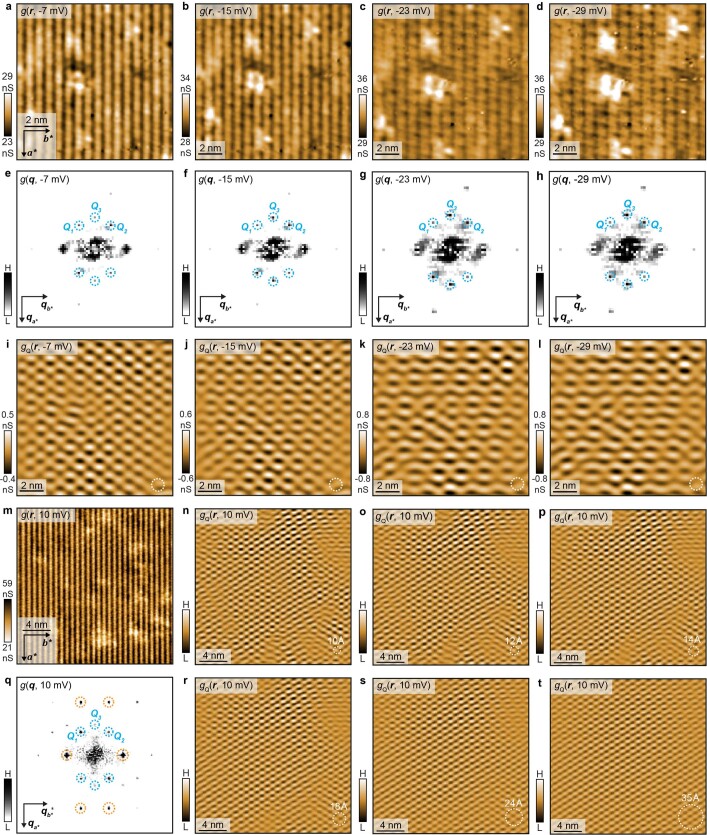
CDW at different voltages in UTe_2_. **a**–**d**, Measured *g*(**r**, *V*) images of UTe_2_ at *T* = 4.2 K and at four representative negative sample voltages, −7 mV, −15 mV, −23 mV and −29 mV, in the same 12 nm × 12 nm FOV. **e**–**h**, Fourier transform of the *g*(**r**, *V*) images, *g*(**q**, *V*), at different sample voltages, showing the presence of the three wavevectors corresponding to the CDW order (in dashed blue circles). **i**–**l**, Inverse Fourier transform of the CDW peaks (**Q**_1_, **Q**_2_, **Q**_3_) at different sample voltages. The CDW pattern is independent from the sample voltages for −29 mV < *V* < −7 mV. A dashed white circle indicates *σ* of the Gaussian filter used to isolate CDW peaks in real space. **m**–**t**, Cutoff dependence of inverse Fourier transform. **n**–**p**,**r**–**t**, Inverse Fourier transform of CDW peaks *g*_Q_(**r**, 10 mV) from *g*(**r**, 10 mV) in **m** and *g*(**q**, 10 mV) in **q**. The images of *g*_Q_(**r**, 10 mV) are filtered at different cutoff lengths, 10 Å, 12 Å, 14 Å, 18 Å, 24 Å and 35 Å. The filter size is in the bottom-right corner. *σ*_**r**_ chosen for Fig. 2d is 14 Å.

UTe_2_ crystals typically cleave to show the (0–11) surface^9,24^, a schematic view of which (Fig. 1b) identifies the key atomic periodicities by vectors a* and b*. At temperature *T* = 4.2 K, this surface is visualized using STM and a typical topographic image *T*(**r**) is shown in Fig. 1d, whereas Fig. 1e shows its power spectral density Fourier transform *T*(**q**), with the surface reciprocal-lattice points identified by dashed orange circles. Pioneering STM studies of UTe_2_ by Aishwarya et al.^24^ have recently discovered a CDW state by visualizing the electronic density of states *g*(**r**, *E*) of such surfaces. As well as the standard maxima at the surface reciprocal-lattice points in *g*(**q**, *E*), the Fourier transform of *g*(**r**, *E*), Aishwarya et al. detected three new maxima with incommensurate wavevectors **Q**_1,2,3_, signifying the existence of a CDW state occurring at temperatures up to at least *T* = 10 K. To emulate this, we measure *g*(**r**, *V*) for −25 mV < *V* < 25 mV at *T* = 4.2 K using a non-superconducting tip on the equivalent cleave surface to ref. ^24^. Figure 2a shows a typical topographic image *T*(**r**) of the (0–11) surface measured at 4.2 K. The Fourier transform *T*(**q**) features the surface reciprocal-lattice points labelled by dashed orange circles in Fig. 2a, inset. The simultaneous image *g*(**r**, 10 mV) in Fig. 2b exhibits the typical modulations in *g*(**r**, *V*) and its Fourier transform *g*(**q**, *V*) in Fig. 2c shows the three CDW peaks^24^ at **Q**_1,2,3_ labelled by dashed blue circles. Inverse Fourier filtration of these three maxima only shows the incommensurate CDW state of UTe_2_. Overall, this state consists predominantly of incommensurate charge-density modulations at three (0–11) in-plane wavevectors **Q**_1,2,3_ that occur at temperatures up to at least 10 K (ref. ^24^) and with a characteristic energy scale up to at least ±25 meV (ref. ^24^; Methods and Extended Data Fig. 1).

## Normal-tip PDW detection at NIS gap edge

**Fig. 3:**
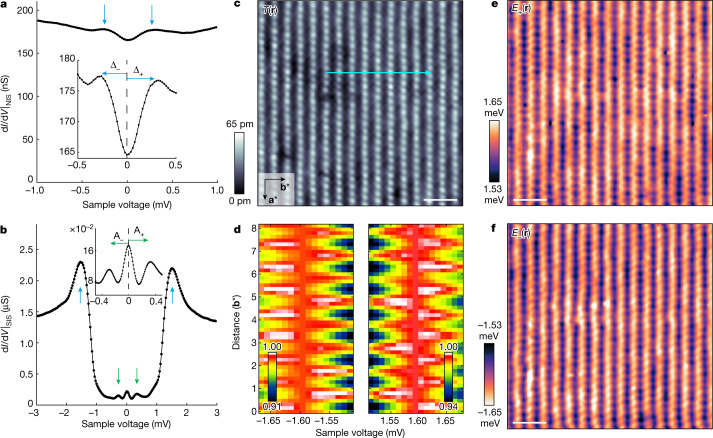
Atomic-resolution imaging of the superconductive energy gap. **a**, Typical NIS spectrum ${\left.{\rm{d}}I/{\rm{d}}V\right|}_{{\rm{NIS}}}$ from normal tip to UTe_2_ (0–11) surface (*I*_s_ = 1 nA, *V*_s_ = −5 mV) at *T* = 280 mK. The inset focuses on the energy range in which the coherence peaks can be detected by conventional normal-tip tunnelling at $E=\pm {\Delta }_{{{\rm{UTe}}}_{{\rm{2}}}}$. Visualizing the superconducting energy gap ${\Delta }_{{{\rm{UTe}}}_{2}}\left({\bf{r}}\right)$ from such ${\left.{\rm{d}}I/{\rm{d}}V\right|}_{{\rm{NIS}}}$ imaging at *T* = 280 mK shows three sets of energy-gap modulations occurring at PDW wavevectors **P**_*i*=1,2,3_ (Methods and Extended Data Fig. 3). We find no deterministic influence of the residual density-of-states modulations on these PDW energy-gap modulations (Methods and Extended Data Fig. 9). **b**, Typical SIS spectrum ${\left.{\rm{d}}I/{\rm{d}}V\right|}_{{\rm{SIS}}}$ from superconducting Nb tip to UTe_2_ (0–11) surface. The blue arrows indicate the convoluted conductance peak located at $\left|{\Delta }_{{\rm{tip}}}+{\Delta }_{{{\rm{UTe}}}_{{\rm{2}}}}\right|$ (*I*_s_ = 3 nA, *V*_s_ = 3 mV). The inset focuses on the energy range in which subgap ${{\rm{d}}I/{\rm{d}}V| }_{{\rm{SIS}}}({\bf{r}},V)$ peaks can be detected at energies *E* = *A*_±_(**r**). **c**, Typical SIS tunnelling topograph *T*(**r**) measured at *T* = 280 mK. Scale bar, 2 nm. **d**, Exemplary normalized ${{\rm{d}}I/{\rm{d}}V| }_{{\rm{SIS}}}({\bf{r}},V)$ focused on the energy ranges near *E*_+_ and *E*_−_ along the trajectory indicated as the light blue arrow in **c**. The modulations of the energies *E*_+_(**r**) and *E*_−_(**r**) of maximum conductance are clearly seen. The two sets of spectra are calibrated such that the ${{\rm{d}}I/{\rm{d}}V| }_{{\rm{SIS}}}(V)$ peaks are particle-hole symmetric. **e**, Measured energy *E*_+_(**r**) at which ${{\rm{d}}I/{\rm{d}}V| }_{{\rm{SIS}}}({V}_{+})$ maxima occur in **c**. The UTe_2_ empty-state superconductive energy gap is ${{\Delta }_{+}\left({\bf{r}}\right)=| E}_{+}\left({\bf{r}}\right)| -| {\Delta }_{{\rm{tip}}}|$, in which $| {\Delta }_{{\rm{tip}}}|$ is a constant. Scale bar, 2 nm. **f**, Measured energy *E*_−_(**r**) at which ${{\rm{d}}I/{\rm{d}}V| }_{{\rm{SIS}}}({V}_{-})$ maxima occur in **c**. The filled-state energy gap is ${{\Delta }_{-}\left({\bf{r}}\right)=| E}_{-}\left({\bf{r}}\right)| -| {\Delta }_{{\rm{tip}}}|$. Scale bar, 2 nm.

Motivated by the discovery that this CDW exhibits an unusual dependence on magnetic field and by the consequent hypothesis that a PDW may exist in this material^24,25^, we next consider direct PDW detection in UTe_2_ by visualizing spatial modulations in its energy gap^17,18,20–23^. The typical tunnelling conductance signature of the UTe_2_ superconducting energy gap is exemplified in Fig. 3a, showing a density-of-states spectrum $N(E={\rm{e}}{\rm{V}})\propto {{\rm{d}}I/{\rm{d}}V|}_{{\rm{N}}{\rm{I}}{\rm{S}}}(V)$ measured using a non-superconducting tip at *T* = 280 mK and junction resistance of *R* ≈ 5 MΩ. Under these circumstances, researchers find only a small drop in the tunnelling conductance at energies $| E| \le | {\Delta }_{{{\rm{UTe}}}_{2}}|$ (ref. ^9^) and concomitantly weak energy maxima in *N*(*E*) at the energy-gap edges $E\approx \pm {\Delta }_{{{\rm{UTe}}}_{{\rm{2}}}}$ (Fig. 3a, inset). Hence, it is challenging to accurately determine the precise value of the energy gap ${\Delta }_{{{\rm{UTe}}}_{{\rm{2}}}}$ (Methods and Extended Data Fig. 3). Nevertheless, we fit a second-order polynomial to the two energy maxima in measured *N*(*E*, **r**) surrounding $E\approx \pm {\Delta }_{{{\rm{UTe}}}_{{\rm{2}}}}$, evaluate the images Δ_±_(**r**) of these energies and then derive a gap map for UTe_2_ as ${\Delta }_{{{\rm{U}}{\rm{T}}{\rm{e}}}_{2}}({\bf{r}})\equiv [{\Delta }_{+}({\bf{r}})-{\Delta }_{-}({\bf{r}})]/2$. Its Fourier transform ${\Delta }_{{{\rm{UTe}}}_{{\rm{2}}}}\left({\bf{q}}\right)$ presented in Methods and Extended Data Fig. 3 shows three incommensurate energy-gap modulations occurring at wavevectors **P**_*i*=1,2,3_, consistent with the wavevectors of the CDW modulations discovered in ref. ^24^. Although this evidence of three PDW states in UTe_2_ is encouraging, its weak signal-to-noise ratio owing to the shallowness of coherence peaks implies that conventional ${\left.{\rm{d}}I/{\rm{d}}V\right|}_{{\rm{NIS}}}$ spectra are inadequate for precision application of equation (6) in this material.

## Superconductive-tip PDW detection

We turn to a well-known technique for improving the resolution of energy maxima in *g*(**r**, *E*) measurements. By using SIS tunnelling from a tip exhibiting high sharp conductance peaks, one can profoundly enhance energy resolution for quasiparticles^26–31^. Most recently, this has been demonstrated in electronic fluid flow visualization^29^ microscopy, with effective energy resolution *δE* ≈ 10 μeV. The SIS current *I* from a superconducting tip is given by the convolution

7 $$I(V)\propto {\int }_{o}^{eV}{N}_{{\rm{t}}{\rm{i}}{\rm{p}}}(E-eV){N}_{{\rm{s}}{\rm{a}}{\rm{m}}{\rm{p}}{\rm{l}}{\rm{e}}}(E){\rm{d}}E$$

Equation (7) demonstrates that using a superconductive tip with high sharp coherence peaks at *E*_±_ = ±Δ_tip_ in *N*_tip_(*E*) will, through convolution, strongly enhance the resolution for measuring the energies ±Δ_sample_ at which energy maxima occur in *N*_sample_(*E*); it will also shift the energy of these features to *E* = ±[Δ_sample_ + Δ_tip_]. In Fig. 3b, we show the ${\left.{\rm{d}}I/{\rm{d}}V\right|}_{{\rm{SIS}}}$ spectrum of a UTe_2_ single crystal using a superconducting Nb tip at *T* = 280 mK. Because the tunnelling current is given by equation (7), the clear maxima in ${\left.{\rm{d}}I/{\rm{d}}V\right|}_{{\rm{SIS}}}$ occur at energies ±(Δ_tip_ + Δ_sample_). With this technique, the energy maxima can be identified with resolution better than *δE* ≤ 10 μeV when *T* < 300 mK (ref. ^29^). Here we use it to improve the signal-to-noise ratio of the UTe_2_ superconductive energy-gap modulations that are already detectable by conventional techniques (Methods and Extended Data Fig. 3).

The UTe_2_ samples are cooled to *T* = 280 mK, with *T*(**r**, *V*) of the (0–11) cleave surface as measured by a superconductive Nb tip shown in Fig. 3c. Here we see a powerful enhancement in the amplitude and sharpness of maxima in ${\left.{\rm{d}}I/{\rm{d}}V\right|}_{{\rm{SIS}}}$ relative to Fig. 3a. Consequently, to determine the spatial arrangements of the energy of the two maxima *E*_+_(**r**) and *E*_−_(**r**) surrounding 1.6 meV exemplified in Fig. 3b, we make two separate *g*(**r**, *V*) maps in the sample bias voltage ranges −1.68 mV < *V* < −1.48 mV and 1.5 mV < *V* < 1.7 mV, and in the identical field of view (FOV). The sharp peak of each ${\left.{\rm{d}}I/{\rm{d}}V\right|}_{{\rm{SIS}}}$ is fit to a second-order polynomial ${\left.{\rm{d}}I/{\rm{d}}V\right|}_{{\rm{SIS}}}=a{V}^{2}+bV+c$, achieving typical quality of fit *R*^2^ = 0.99 ± 0.005. The energy of maximum intensity in *E*_+_(**r**) or *E*_−_(**r**) is then identified analytically from the fit parameters (Methods and Extended Data Fig. 4). The fine line across Fig. 3c specifies the trajectory of an exemplary series of ${\left.{\rm{d}}I/{\rm{d}}V\right|}_{{\rm{SIS}}}$ spectra, whereas Fig. 3d presents the colour map ${\left.{\rm{d}}I/{\rm{d}}V\right|}_{{\rm{SIS}}}$ spectra for both positive and negative energy coherence peaks along this line. Periodic variations in the energies at which pairs of peaks occur are obvious, directly demonstrating that *E*_+_(**r**) and *E*_−_(**r**) are modulating periodically but in energetically opposite directions. Using this *g*(***r***, *V*) measurement and fitting procedure (Methods and Extended Data Fig. 4) yields atomically resolved images of *E*_+_(**r**) and *E*_−_(**r**). The magnitude of both positive and negative superconductive energy gaps of UTe_2_ is then ${\Delta }_{\pm }\left({\bf{r}}\right)\equiv \left|{E}_{\pm }\left({\bf{r}}\right)\right|-{\rm{| }}{\Delta }_{{\rm{tip}}}|$, in which |Δ_tip_| is constant. These two independently measured gap maps Δ_+_(**r**) and Δ_−_(**r**) are spatially registered to each other at every location with 27-pm precision so that the cross-correlation coefficient between them is *X* ≅ 0.92, meaning that the superconducting energy-gap modulations are entirely particle-hole symmetric (Fig. 3e,f, Methods and Extended Data Fig. 5).

**Fig. 4:**
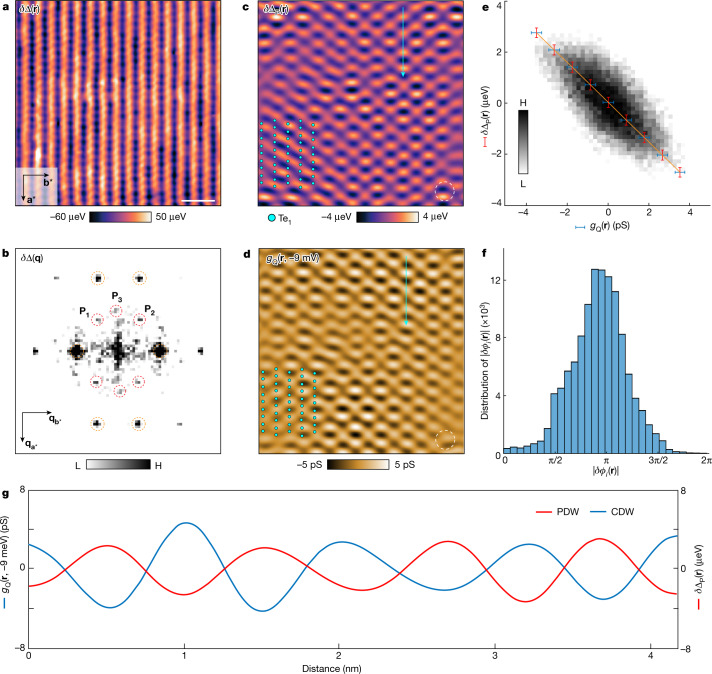
Visualizing the PDW state of UTe_2_. **a**, Measured variations in energy gap *δ*Δ(**r**) from Fig. 3c. Scale bar, 2 nm. **b**, Measured *δ*Δ(**q**) from **a**. The surface reciprocal-lattice points are labelled by dashed orange circles and the PDW peaks at **P**_1,2,3_ are labelled by dashed red circles. **P**_1,2,3_ are linked by reciprocal-lattice vectors (Extended Data Fig. 10). *δ*Δ(**r**) and *δ*Δ(**q**) exhibit superior signal-to-noise ratio as compared with the normal-tip gap map ${\Delta }_{{{\rm{UTe}}}_{{\rm{2}}}}\left({\bf{r}}\right)$ (Extended Data Fig. 8). **c**, Inverse Fourier transform filtered *δ*Δ(**q**) of panel **a** at **P**_1,2,3_ shows the first visualization of the PDW (filter size is 11.4 Å). The PDW is repeatable in experimental measurements (Extended Data Fig. 6) and also independently evidenced in Methods and Extended Data Fig. 7. **d**, Image of *g*_Q_(**r**, −9 mV) of the CDW, measured at *T* = 4.2 K in the same FOV as panel **c** from inverse Fourier transform filtered *g*(**r**, −9 mV) at **Q**_1,2,3_ (filter size is 11.4 Å). The precision of registration between the CDW and PDW images is 27 pm (Methods and Extended Data Fig. 5). These coincident CDW and PDW images are measured in the energy ranges 10 meV and 10 μeV, respectively, and appear visually distinct, yet their cross-correlation coefficient of −0.68 shows their anticorrelation. The CDW maxima exist at the PDW minima. **e**, Statistical relationship between *δ*Δ_P_(**r**) and *g*_Q_(**r**, −9 mV). The *δ*Δ_P_(**r**) and *g*_Q_(**r**, −9 mV) are strongly anticorrelated spatially. They are approximately negatives of each other. **f**, Statistics of the relative spatial phase difference *δϕ*_*i*_ between the CDW phase ${\phi }_{i}^{{\rm{C}}}\left({\bf{r}}\right)$ at **Q**_*i*_ and the PDW phase ${\phi }_{i}^{{\rm{P}}}\left({\bf{r}}\right)$ at **P**_*i*_ in the coterminous images *g*_Q_(**r**, −9 mV) and *δ*Δ_P_(**r**). The spatial phase difference, defined as $| \delta {{\phi }}_{i}\left({\bf{r}}\right)| \equiv | {{\phi }}_{i}^{{\rm{P}}}\left({\bf{r}}\right)-{{\phi }}_{i}^{{\rm{C}}}\left({\bf{r}}\right)|$, between all three CDWs and PDWs at **Q**_*i*_:**P**_*i*_ is ${\left|\delta \phi \right|}_{{\rm{RMS}}}=0.96{\rm{\pi }}$. **g**, Coterminous measurement of CDW *g*_Q_(**r**, −9 mV) and PDW *δ*Δ_P_(**r**) along a trajectory (arrows in panels **c** and **d**).

From these and equivalent data, the UTe_2_ superconducting energy-gap structure ${\Delta }_{{{\rm{UTe}}}_{2}}\left({\bf{r}}\right)=\left({\Delta }_{+}\left({\bf{r}}\right)+{\Delta }_{-}\left({\bf{r}}\right)\right)/2$ can now be examined for its spatial variations *δ*Δ(**r**) by using

8 $$\delta \Delta ({\bf{r}})\equiv {\Delta }_{{{\rm{UTe}}}_{{\rm{2}}}}\left({\bf{r}}\right)-\left\langle {\Delta }_{{{\rm{UTe}}}_{{\rm{2}}}}\left({\bf{r}}\right)\right\rangle $$

in which $\left\langle {\Delta }_{{{\rm{UTe}}}_{{\rm{2}}}}\left({\bf{r}}\right)\right\rangle$ is the spatial average over the whole FOV. Figure 4a shows measured *δ*Δ(**r**) in the same FOV as Fig. 3c. The Fourier transform of *δ*Δ(**r**), *δ*Δ(**q**), is presented in Fig. 4b, in which the surface reciprocal-lattice points are identified by dashed orange circles. The three further peaks labelled by dashed red circles represent energy-gap modulations with incommensurate wavevectors at **P**_1,2,3_ of the PDW state in UTe_2_. Focusing only on these three wavevectors **P**_1,2,3_, we perform an inverse Fourier transform to show the spatial structure of the UTe_2_ PDW state in Fig. 4c (Methods). This state seems to consist predominantly of incommensurate superconductive energy-gap modulations at three (0–11) in-plane wavevectors **P**_1,2,3_ with a characteristic energy scale 10 μeV for peak-to-peak modulations.

## Energy modulations of Andreev resonances

There is an alternative modality of SIS tunnelling, namely, measuring the effects of Andreev reflections. For two superconductors with very different gap magnitudes, when the sample bias voltage shifts the smaller gap edge (UTe_2_ in this case) to the chemical potential of the other superconductor, the Andreev process of electron (hole) transmission and hole (electron) reflection plus electron-pair propagation can produce an energy maximum in d*I*/d*V*|_SIS_ (ref. ^38^), an effect well attested by experiment^39^. Here, by imaging the signed energies of *A*_±_(**r**) of two subgap d*I*/d*V*|_SIS_ maxima detected throughout our studies and identified by the green arrows in Fig. 3b, an Andreev-resonance measure of the UTe_2_ energy gap is conjectured as ${\Delta }_{{\rm{A}}}({\bf{r}})\equiv \left[{A}_{+}({\bf{r}})-{A}_{-}({\bf{r}})\right]/2$. These data are presented in Methods and Extended Data Fig. 7 and show a Δ_A_(**r**) modulating with amplitude approximately 10 μeV at wavevectors **P**_1,2_ state, further evidencing the UTe_2_ PDW state.

## Visualizing the interplay of PDW and CDW

Finally, one may consider the two cases of intertwining outlined earlier: (1) Δ_S_(**r**) and Δ_P_(**r**) are predominant and generate charge modulations ${\rho }_{{\rm{P}}}\left({\bf{r}}\right)\propto {\Delta }_{{\rm{S}}}^{* }{\Delta }_{{\rm{P}}}+{\Delta }_{-{\rm{P}}}^{* }{\Delta }_{{\rm{S}}}$ and ${\rho }_{2{\rm{P}}}\left({\bf{r}}\right)\propto {{\Delta }_{-{\rm{P}}}^{* }\Delta }_{{\rm{P}}}$ or (2) Δ_S_(**r**) and *ρ*_Q_(**r**) are predominant and generate pair density modulations ${\Delta }_{{\rm{Q}}}\left({\bf{r}}\right)\propto {\Delta }_{{\rm{S}}}^{* }{\rho }_{{\rm{Q}}}$. For case (1) to be correct here, a PDW with magnitude 10 μeV coexisting with a superconductor of gap maximum near 250 μeV must generate a CDW on the energy scale 25 meV and exist up to at least *T* = 10 K. For case (2) to be valid, a normal-state CDW with eigenstates at energies up to 25 meV coexisting with a superconductor of gap magnitude 250 μeV must generate a PDW at the same wavevector and with amplitude near 10 μeV. Intuitively, the latter case seems the most plausible for UTe_2_.

To explore this issue further, we visualize the CDW in the non-superconductive state at *T* = 4.2 K, then cool to *T* = 280 mK and visualize the PDW in precisely the same FOV. Figure 4c,d shows the result of such an experiment in the FOV of Fig. 3c. The CDW and PDW images are registered to the underlying lattice and to each other with 27-pm precision. Comparing their coterminous images in Fig. 4c and Fig. 4d shows that the CDW and PDW states of UTe_2_ appear spatially distinct. Yet, they are actually registered to each other in space, being approximate negative images of each other (Fig. 4e) and with a measured relative phase for all three ***P***_*i*_:***Q***_*i*_ pairs of $| \delta {\phi }_{i}| \cong \pi$ (Fig. 4f, Methods and Extended Data Fig. 10). A typical example of this effect is shown in a line cut across Fig. 4c,d along the Te chain direction, with the directly measured values shown in Fig. 4g. The direct and comprehensive knowledge of CDW and PDW characteristics and interactions presented in Fig. 4 now motivates search for a Ginzburg–Landau description capable of capturing this complex intertwined phenomenology and that reported in ref. ^24^.

## Conclusions

Notwithstanding such theoretical challenges, in this study, we have demonstrated that PDWs occur at three incommensurate wavevectors **P**_*i*=1,2,3_ on the (0–11) surface of UTe_2_ (Fig. 4b,c). These wavevectors are indistinguishable from the wavevectors **Q**_*i*=1,2,3_ of the prevenient normal-state CDW at the equivalent surface (Figs. 2c and 4d). All three PDWs exhibit peak-to-peak gap energy modulations in the range near 10 μeV (Fig. 4c,g). When the **P**_*i*=1,2,3_ PDW states are visualized at 280 mK in the identical FOV as the **Q**_*i*=1,2,3_ CDWs visualized above the superconductive *T*_c_, every **Q**_*i*_:**P**_*i*_ pair is spatially registered to each other (Fig. 4c,d), but with a relative phase shift of $| \delta {\phi }_{i}\,| \cong \pi$ throughout (Fig. 4f). Given the premise that UTe_2_ is a spin-triplet superconductor^12^, the PDW phenomenology detected and described herein (Fig. 4) signifies the entrée to spin-triplet PDW physics.

## Methods

### CDW visualization in non-superconductive UTe_2_

#### Differential conductance imaging of CDW at *T* = 4.2 K

At *T* = 4.2 K and using superconducting tips to study the UTe_2_ (0–11) surface, we measure differential tunnelling conductance spectra *g*(***r***, *V*) to visualize the CDW in the normal state of UTe_2_. Extended Data Fig. 1a–d shows *g*(**r**, *V*) images *V* = −7 mV, −15 mV, −23 mV and −29 mV with Fourier transform *g*(**q**, *V*) shown as Extended Data Fig. 1e–h. Three CDW peaks at **Q**_1,2,3_ occur in all *g*(**q**, *V*), representing incommensurate charge-density modulations with energy scale up to approximately 30 meV, consistent with ref. ^24^.

#### CDW visualization at incommensurate wavevectors **Q**_1,2,3_

To calculate the amplitude ${g}_{{{\bf{Q}}}_{i}}({\bf{r}})$ of the CDW modulation represented by the peaks at **Q**_*i*_ (*i* = 1, 2, 3), we apply a two-dimensional computational lock-in technique. Here *g*(**r**) is multiplied by the term ${{\rm{e}}}^{{\rm{i}}{{\bf{Q}}}_{i}\cdot {\bf{r}}}$ and integrated over a Gaussian filter to obtain the lock-in signal

9 $${g}_{{{\bf{Q}}}_{i}}\left({\bf{r}}\right)=\frac{1}{\sqrt{2\pi }{\sigma }_{{\bf{r}}}}\int {\rm{d}}{\bf{R}}g\left({\bf{R}}\right){{\rm{e}}}^{{\rm{i}}{{\bf{Q}}}_{i}\cdot {\bf{R}}}{{\rm{e}}}^{\frac{{\left|{\bf{r}}-{\bf{R}}\right|}^{2}}{2{\sigma }_{{\bf{r}}}^{2}}}$$

in which *σ*_**r**_ is the cutoff length in the real space. In **q** space, this lock-in signal is

10 $${g}_{{{\bf{Q}}}_{i}}({\bf{r}})={{\mathscr{F}}}^{-1}{g}_{{{\bf{Q}}}_{i}}({\bf{q}})={{\mathscr{F}}}^{-1}\,\left[{\mathscr{F}}(\,g({\bf{r}}){{\rm{e}}}^{{\rm{i}}{{\bf{Q}}}_{i}\cdot {\bf{r}}})\cdot \frac{1}{\sqrt{2\pi }{\sigma }_{{\bf{Q}}}}{{\rm{e}}}^{-\frac{{q}^{2}}{2{{\sigma }_{{\bf{Q}}}}^{2}}}\right]$$

in which *σ*_**Q**_ is the cutoff length in **q** space. Here ${{\sigma }_{{\bf{r}}}=1/\sigma }_{{\bf{Q}}}$. Next, *g*_Q_(**r**, *V*), the inverse Fourier transform of the combined **Q**_*i*_ (*i* = 1, 2, 3) CDWs, is presented in Extended Data Fig. 1i–l.

To specify the effect of filter size on the inverse Fourier transform, we show in Extended Data Fig. 1m–t the real-space density of states *g*(**r**, 10 mV), its Fourier transform *g*(**q**, 10 mV) and the evolution of inverse Fourier transform images as a function of the real-space cutoff length *σ*_**r**_. The differential conductance map *g*_Q_(**r**, 10 mV) is shown at a series of *σ*_**r**_, including 10 Å, 12 Å, 14 Å, 18 Å, 24 Å and 35 Å. The distributions of the CDW domains in the filtered *g*_Q_(**r**, 10 mV) images with cutoff lengths of 10 Å, 12 Å, 14 Å, 18 Å and 24 Å are highly similar. The cutoff length used in Fig. 2d is 14 Å, such that the domains of the CDW modulations are resolved and the irrelevant image distortions are excluded. The same filter size of 14 Å is chosen for all three **Q**_*i*_ vectors. Formally, the equivalent inverse Fourier transform analysis is carried out for Fig. 4c,d but with a filter size of 11.4 Å to filter both the CDW and PDW peaks.

#### Simulated UTe_2_ topography

**Extended Data Fig. 2:**
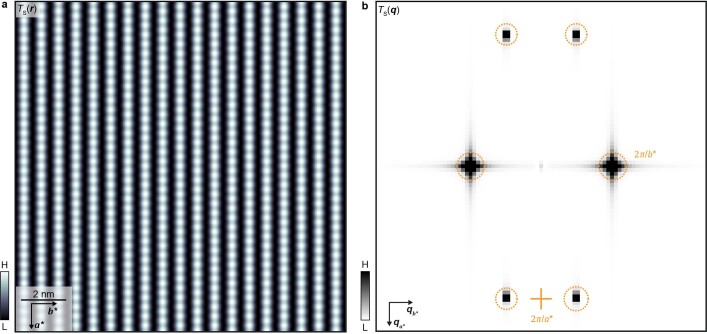
Simulated topography of UTe_2_ and its Fourier transform. **a**, Simulated topograph, *T*_S_(**r**) of the (0–11) cleave surface of UTe_2_. **b**, Fourier transform of simulated topograph, *T*_S_(**q**). The six primary peaks occur at the reciprocal-lattice wavevectors and are observed in the experimental STM data.

To identify **q**-space peaks resulting from the (0–11) cleave-plane structure of UTe_2_, we simulate the topography of the UTe_2_ cleave plane and Fourier transform. Subsequently, we can distinguish clearly the CDW signal from the structural periodicity of the surface. The simulation is calculated on the basis of the ideal lattice constant of the (0–11) plane of the UTe_2_, a* = 4.16 Å and inter-Te-chain distance b* = 7.62 Å. Extended Data Fig. 2a is a simulated *T*(**r**) image in the FOV of 14.5 nm. The simulated topography *T*(**r**) is in good agreement with experimental *T*(**r**) images presented throughout. The Fourier transform, *T*(**q**), of the simulated *T*(**r**) in Extended Data Fig. 2b shows six sharp peaks, confirming that they are the primary peaks resulting from the cleave-plane structure. Most notably, the CDW peaks in Fig. 2c are not seen in the simulation. They are therefore not caused by the surface periodicity but instead originate from the electronic structure, as first demonstrated in ref. ^24^.

### Normal-tip PDW detection at the NIS gap edge of UTe_2_

**Extended Data Fig. 3:**
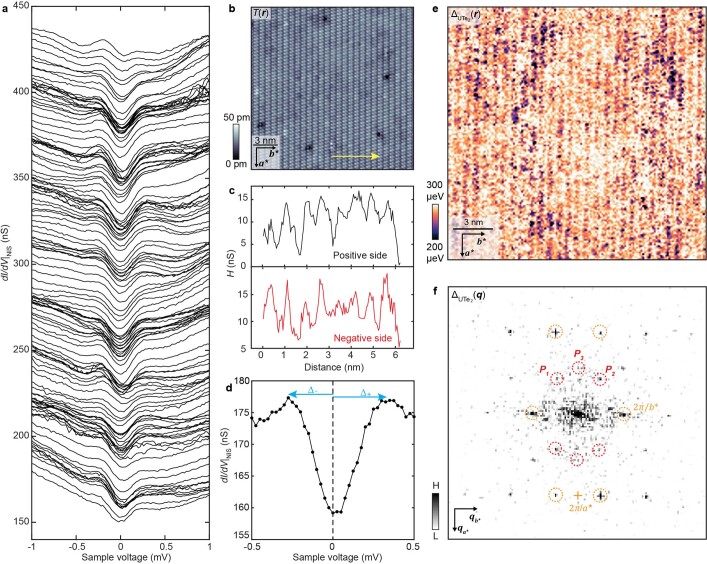
PDW detection using a normal tip. **a**, A typical line cut of ${\rm{d}}I\,/\,{\rm{d}}V{| }_{{\rm{NIS}}}$ spectra obtained at 280 mK along the trajectory shown in **b** (*I*_s_ = 1 nA, *V*_s_ = −5 mV). **b**, Topograph *T*(**r**) obtained using a normal tip. **c**, Gap depth *H* distribution along the trajectory in **b**. **d**, ${\rm{d}}I\,/\,{\rm{d}}V{| }_{{\rm{NIS}}}$ spectrum showing the superconducting gap Δ_+_ and Δ_−_. **e**, Image of half the energy difference between superconducting coherence peaks, that is, the superconducting energy gap of ${\Delta }_{{{\rm{UTe}}}_{{\rm{2}}}}({\bf{r}})$, obtained in the same FOV as **b**, using conventional normal-tip imaging. **f**, ${\Delta }_{{{\rm{UTe}}}_{{\rm{2}}}}({\bf{q}})$, the Fourier transform of ${\Delta }_{{{\rm{UTe}}}_{{\rm{2}}}}({\bf{r}})$. Three peaks are seen at the same wavevector as the normal-state CDW and indicate the existence of three superconducting PDW states (*I*_s_ = 1 nA, *V*_s_ = −5 mV).

Initial STM searches for a PDW on UTe_2_ were carried out using a normal tip at 280 mK. Extended Data Fig. 3a shows a typical line cut of the ${\rm{d}}I\,/\,{\rm{d}}V{| }_{{\rm{NIS}}}$ spectrum taken from the FOV in Extended Data Fig. 3b. There is a large residual density of states near the Fermi level. The gap depth *H* is defined as the difference between the gap bottom in the ${\rm{d}}I\,/\,{\rm{d}}V{| }_{{\rm{NIS}}}$ spectrum and the coherence peak height, that is, $H\equiv {\rm{d}}I\,/\,{\rm{d}}V\,{{\rm{| }}}_{{\rm{NIS}}}(V\equiv {\Delta }_{{{\rm{UTe}}}_{{\rm{2}}}})-{\rm{d}}I\,/\,{\rm{d}}V\,{{\rm{| }}}_{{\rm{NIS}}}(V\equiv 0)$. Its modulation is extracted from the ${\rm{d}}I\,/\,{\rm{d}}V{| }_{{\rm{NIS}}}$ line cut and presented in Extended Data Fig. 3c; it modulates perpendicular to the Te atom chains.

Conventional, NIS tunnelling discloses superconducting energy-gap modulations as shown in Extended Data Fig. 3a. The superconducting energy gap is defined as half of the peak-to-peak distance in the ${\rm{d}}I\,/\,{\rm{d}}V\,{{\rm{| }}}_{{\rm{NIS}}}$ spectrum (Fig. 3a and Extended Data Fig. 3d). Its magnitude $| {\Delta }_{{{\rm{UTe}}}_{{\rm{2}}}}|$ is found to lie approximately between 250 µeV and 300 µeV. We measure variations in the coherence peak position from the ${\rm{d}}I\,/\,{\rm{d}}V{| }_{{\rm{NIS}}}$ spectrum at each location **r**. The two energy maxima near ${\Delta }_{{{\rm{UTe}}}_{{\rm{2}}}}$ of each ${\rm{d}}I\,/\,{\rm{d}}V\,{{\rm{| }}}_{{\rm{NIS}}}$ spectrum are fitted with a second-order polynomial function (${R}_{{\rm{RMS}}}^{2}=0.87$). The energy gap is defined as the maxima of the fit, Δ_+_ for *V* > 0 and Δ_−_ for *V* < 0. The total gap map ${\Delta }_{{{\rm{UTe}}}_{{\rm{2}}}}({\bf{r}})\equiv [{\Delta }_{+}({\bf{r}})-{\Delta }_{-}({\bf{r}})]/2$ is derived from Δ_+_ and Δ_−_ (Extended Data Fig. 3e). The Fourier transform of ${\Delta }_{{{\rm{UTe}}}_{{\rm{2}}}}\left({\bf{r}}\right)$, ${\Delta }_{{{\rm{UTe}}}_{{\rm{2}}}}\left({\bf{q}}\right)$ (Extended Data Fig. 3f), shows three peaks at wavevectors **P**_*i*=1,2,3_. They are the initial signatures of the energy-gap modulations of the three coexisting PDW states in UTe_2_.

### Superconductive-tip PDW visualization at the SIS gap edge of UTe_2_

#### Tip preparation

**Extended Data Fig. 4:**
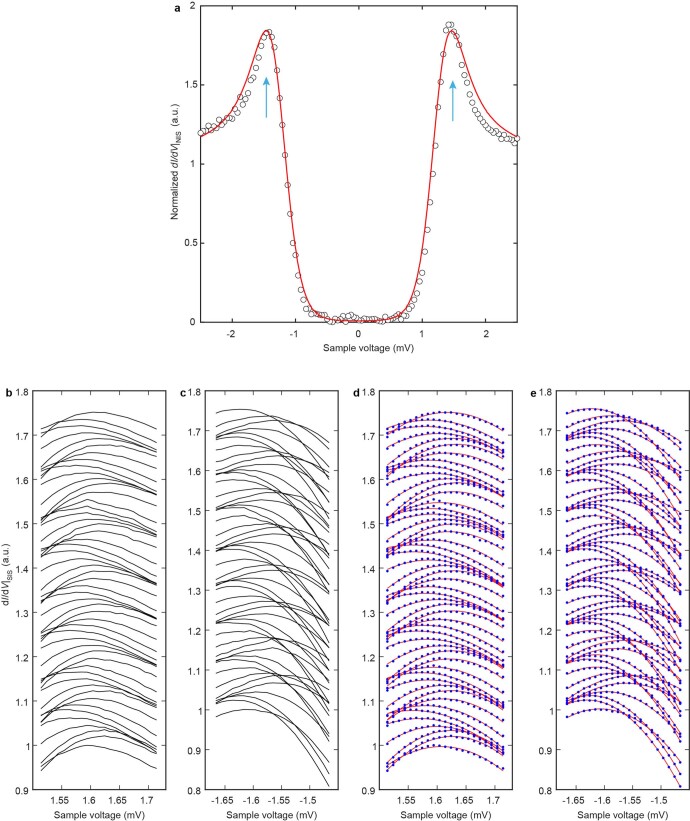
Determination of the tip gap Δ_tip_ and evolution of d*I*/d*V*|_SIS_ spectra with parabolic fitting. **a**, A typical spectrum measured on UTe_2_ using a superconducting Nb tip at 1.5 K (*I*_s_ = 100 pA, *V*_s_ = 4 mV). At this temperature, the UTe_2_ gap is closed, thus the coherence peak value shows the pure Nb tip gap of 1.37 meV. The spectrum is clearly well fitted using the Dynes model. The fitting parameters of the Dynes model are Γ = 0.01 meV, Δ = 1.37 meV. **b**,**c**, Line cuts of ${\rm{d}}I\,/\,{\rm{d}}V{| }_{{\rm{SIS}}}\left(V\right)$ spectra measured at both negative bias and positive bias along the trajectory shown in Fig. 3c. **d**,**e**, The evolution of ${\rm{d}}I\,/\,{\rm{d}}V{| }_{{\rm{SIS}}}\left(V\right)$ spectra (blue points) from the same data shown in **b** and **c** and their parabolic fits *g*(*V*).

Atomic-resolution Nb superconducting tips are prepared by field emission. To determine the tip gap value during our experiments, we measure conductance spectrum on UTe_2_ at 1.5 K (*T*_c_ = 1.65 K), in which the UTe_2_ superconducting gap is closed. The tip gap $| {\Delta }_{{\rm{tip}}}| \cong 1.37\,{\rm{meV}}$ is the energy of the coherence peak (Extended Data Fig. 4a). The measured spectrum is fitted using a Dynes model^40^. The typical ${\rm{d}}I\,/\,{\rm{d}}V{| }_{{\rm{SIS}}}$ measured at 280 mK on UTe_2_ (Fig. 3b) shows the total gap value $E=| {\Delta }_{{\rm{tip}}}| +| {\Delta }_{{{\rm{UTe}}}_{{\rm{2}}}}| \approx 1.62\,{\rm{meV}}$. Therefore, we estimate $| {\Delta }_{{{\rm{UTe}}}_{{\rm{2}}}}| \approx 0.25\,{\rm{meV}}$, consistent with a previous report^9^ and the ${\rm{d}}I\,/\,{\rm{d}}V{| }_{{\rm{NIS}}}$ shown in Fig. 3a and Extended Data Fig. 3.

#### SIS tunnelling amplification of energy-gap measurements

To determine the energy of *E*_+_(**r**) and *E*_−_(**r**) at which the maximum conductance in ${\rm{d}}I\,/\,{\rm{d}}V{| }_{{\rm{SIS}}}(V)$ occurs, we fit the peak of the measured ${\rm{d}}I\,/\,{\rm{d}}V{| }_{{\rm{SIS}}}(V)$ spectra using a second-order polynomial fit:

11 $$g(V)=a{V}^{2}+bV+c$$

This polynomial closely fits the experimental data. Extended Data Fig. 4b,c shows two typical ${\rm{d}}I\,/\,{\rm{d}}V{| }_{{\rm{SIS}}}(V)$ spectra measured at +*V* and −*V* along the trajectory indicated in Fig. 3c. The evolution of fits *g*(*V*) in Extended Data Fig. 4d,e shows a very clear energy-gap modulation.

#### Shear correction and Lawler–Fujita algorithm

To register several images to precisely the same FOV, the Lawler–Fujita algorithm is applied to the experimental data. Then, to recover the arbitrary hexagon of the Te lattice, shear correction is applied to correct any image distortions caused by long-range scanning drift during days of continuous measurement.

To correct against picometre-scale distortions of the lattice, we apply the Lawler–Fujita algorithm. Let $\widetilde{T}(\widetilde{{\bf{r}}})$ represent a topograph of a perfect UTe_2_ lattice without any distortion. Three pairs of Bragg peaks **Q**_1_, **Q**_2_ and **Q**_3_ can be obtained from Fourier transform of $\widetilde{T}(\widetilde{{\bf{r}}})$. Hence $\widetilde{T}(\widetilde{{\bf{r}}})$ is expected to take the form

12 $$\widetilde{T}(\widetilde{{\bf{r}}})=\mathop{\sum }\limits_{i=1}^{3}{T}_{i}\cos \left({{\bf{Q}}}_{i}\cdot \widetilde{{\bf{r}}}+{\theta }_{i}\right)$$

The experimentally obtained topography *T*(**r**) may suffer from a slowly varying position-dependent spatial phase shift *θ*_*i*_(**r**), which can be given by

13 $$T({\bf{r}})=\mathop{\sum }\limits_{i=1}^{3}{T}_{i}\cos \left({{\bf{Q}}}_{i}\cdot {\bf{r}}+{\theta }_{i}({\bf{r}})\right)$$

To get *θ*_*i*_(**r**), we use a computational two-dimensional lock-in technique to the topography

14 $${A}_{{\bf{Q}}}\,({\bf{r}})=\int {\rm{d}}{\bf{R}}T({\bf{R}}){{\rm{e}}}^{{\rm{i}}{\bf{Q}}\cdot {\bf{R}}}{{\rm{e}}}^{-\frac{{\left({\bf{r}}-{\bf{R}}\right)}^{2}}{2{\sigma }^{2}}}$$

15 $${A}_{{{\bf{Q}}}_{i}}\left(\,{\bf{r}}\right)={{\mathscr{F}}}^{-1}{A}_{{{\bf{Q}}}_{i}}\left(\,{\bf{q}}\right)={{\mathscr{F}}}^{-1}\,\left[{\mathscr{F}}(T\left({\bf{r}}\right){{\rm{e}}}^{{\rm{i}}{{\bf{Q}}}_{i}\cdot {\bf{r}}})\cdot \frac{1}{\sqrt{2\pi }{\sigma }_{{\rm{Q}}}}{{\rm{e}}}^{-\frac{{q}^{2}}{2{{\sigma }_{{\rm{Q}}}}^{2}}}\right]$$

16 $$\left|{A}_{{\bf{Q}}}\left(\,{\bf{r}}\right)\right|=\sqrt{{\left({\rm{Re}}{A}_{{\bf{Q}}}\left({\bf{r}}\right)\right)}^{2}+{\left({\rm{Im}}{A}_{{\bf{Q}}}\left({\bf{r}}\right)\right)}^{2}}$$

17 $${\theta }_{i}({\bf{r}})={\tan }^{-1}\frac{{\rm{Im}}{A}_{{\bf{Q}}}\left(\,{\bf{r}}\right)}{{\rm{Re}}{A}_{{\bf{Q}}}\left(\,{\bf{r}}\right)}$$

for which *σ* is chosen to capture the lattice distortions. In the Lawler–Fujita analysis, we use *σ*_*q*_ = 3.8 nm^−1^. Mathematically, the relationship between the distorted and the perfect lattice for each **Q**_*i*_ is ${{\bf{Q}}}_{i}\cdot {\bf{r}}{\boldsymbol{+}}{\theta }_{i}({\bf{r}})={{\bf{Q}}}_{i}\cdot \widetilde{{\bf{r}}}+{\theta }_{i}$. We define another global-position-dependent quantity, the displacement field ${\bf{u}}\left({\bf{r}}\right)={\bf{r}}-\widetilde{{\bf{r}}}$, which can be obtained by solving equations

18 $${\bf{u}}\left({\bf{r}}\right)={\left(\begin{array}{c}\begin{array}{c}{{\bf{Q}}}_{1}\\ {{\bf{Q}}}_{2}\end{array}\\ {{\bf{Q}}}_{3}\end{array}\right)}^{-1}\left(\begin{array}{c}\begin{array}{c}{\theta }_{1}-{\theta }_{1}\left({\bf{r}}\right)\\ {\theta }_{2}-{\theta }_{2}\left({\bf{r}}\right)\end{array}\\ {\theta }_{3}-{\theta }_{3}\left({\bf{r}}\right)\end{array}\right)$$

Finally, a drift-corrected topography, $\widetilde{T}\left(\widetilde{{\bf{r}}}\right)$ is obtained by

19 $$\widetilde{T}\left(\widetilde{{\bf{r}}}\right)=T({\bf{r}}-{\bf{u}}\left({\bf{r}}\right))$$

By applying the same correction of **u**(**r**) to the simultaneously taken differential conductance map *g*(**r**), we can get

20 $$\widetilde{g}\left(\widetilde{{\bf{r}}}\right)=g({\bf{r}}-{\bf{u}}({\bf{r}}))$$

in which $\widetilde{g}\left(\widetilde{{\bf{r}}}\right)$ is the drift-corrected differential conductance map.

#### Lattice registration of UTe_2_ energy gap ${{\boldsymbol{\Delta }}}_{{{\bf{UTe}}}_{{\bf{2}}}}{\boldsymbol{(}}{\bf{r}}{\boldsymbol{)}}$

We measure two separate ${\rm{d}}I\,/\,{\rm{d}}V{| }_{{\rm{SIS}}}({\bf{r}},V)$ maps separated by several days and in two overlapping FOVs, with energy ranges −1.68 meV < *E* < −1.48 meV and 1.5 meV < *E* < 1.7 meV. Therefore, we obtain two datasets, *T*_+_(**r**) with the simultaneous ${{{\rm{d}}I/{\rm{d}}V| }_{{\rm{SIS}}}}_{+}({\bf{r}},V)$ at positive bias and *T*_−_(**r**) with the simultaneous ${{{\rm{d}}I/{\rm{d}}V| }_{{\rm{SIS}}}}_{-}({\bf{r}},V)$ at negative bias.

**Extended Data Fig. 5:**
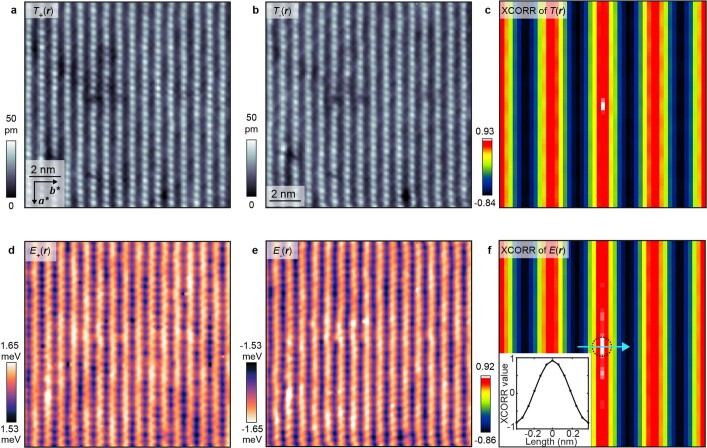
Spatial registration of topographs and gap maps. **a**,**b**, 12 nm × 12 nm topographs after registration. These topographs were obtained concomitantly as ${\rm{d}}I\,/\,{\rm{d}}V{| }_{{\rm{SIS}}}\left({\bf{r}},V\right)$ maps recording positive and negative coherence peaks, respectively. **c**, XCORR map of the registered topographs. The correlation coefficient is 0.93, indicating that the two topographs are almost identical. The maxima of the XCORR map is a single pixel wide, which suggests a registration precision of 0.5 pixels, equivalent to registration precision of 27 pm. **d**, Positive coherence peak map *E*_+_(**r**) from **a**. **e**, Negative coherence peak map *E*_−_(**r**) from **b**. **f**, XCORR map providing a two-dimensional measure of correlation between the positive gap map *E*_+_(**r**) and negative gap map *E*_−_(**r**). Inset, a line cut along the trajectory indicated in **f**. It shows that the maximum is 0.92 and coincides with the (0, 0) cross-correlation vector. The strong correlation demonstrates the particle-hole symmetry in superconductive UTe_2_.

After the shear and Lawler–Fujita corrections are applied, the lattice in the corrected topographs of *T*_+_(**r**) and *T*_−_(**r**) become nearly perfectly periodic. Next, we perform rigid spatial translations to register the two topographs to the exact same FOV with a lateral precision better than 27 pm. Extended Data Fig. 5a,b shows two topographs of registered *T*_+_(**r**) and *T*_−_(**r**). Cross-correlation (XCORR) of two images *I*_1_ and *I*_2_, *X*(**r**, *I*_1_, *I*_2_) at **r** is obtained by sliding two images **r** apart and calculating the convolution,

21 $$X({\bf{r}},{I}_{1},{I}_{2})=\frac{\int {I}_{1}^{\ast }({{\bf{r}}}_{1}){I}_{2}({\bf{r}}+{{\bf{r}}}_{1}){\rm{d}}{{\bf{r}}}_{1}}{\sqrt{\int {|{I}_{1}({{\bf{r}}}_{1})|}^{2}{\rm{d}}{{\bf{r}}}_{1}\int {|{I}_{2}({{\bf{r}}}_{2})|}^{2}{\rm{d}}{{\bf{r}}}_{2}}}$$

in which the denominator is a normalization factor such that, when *I*_1_ and *I*_2_ are exactly the same image, we can get *X*(**r** = **0**, *I*_1_, *I*_2_) = 1 with the maximum centred at (0, 0) cross-correlation vector. Extended Data Fig. 5c shows that the maximum of XCORR between *T*_+_(**r**) and *T*_−_(**r**) coincides with the (0, 0) cross-correlation vector. The offset of the two registered images are within one pixel. The multiple-image registration method is better than 0.5 pixels = 27 pm in the whole FOV and the maxima of the cross-correlation coefficient between the topographs is 0.93. All transformation parameters applied to *T*_+_(**r**) and *T*_−_(**r**) to yield the corrected topographs are subsequently applied to the corresponding ${\rm{d}}I\,/\,{\rm{d}}V{| }_{{\rm{SIS}}}({\bf{r}},V)$ maps obtained at positive and negative voltages.

#### Particle-hole symmetry of the superconducting energy gap ${{\boldsymbol{\Delta }}}_{{{\bf{UTe}}}_{{\bf{2}}}}{\boldsymbol{(}}{\bf{r}}{\boldsymbol{)}}$

The cross-correlation map in Extended Data Fig. 5f provides a two-dimensional measure of agreement between the positive and negative ${\rm{d}}I\,/\,{\rm{d}}V{| }_{{\rm{SIS}}}\left(V\right)$ energy-maxima maps in Extended Data Fig. 5d,e. The inset of Extended Data Fig. 5f shows a line cut along the trajectory indicated in Extended Data Fig. 5f. It shows a maximum of 0.92 and coincides with the (0, 0) cross-correlation vector. Thus, it shows that gap values between positive bias and negative bias are highly correlated.

#### PDW visualization at incommensurate wavevectors **P**_1,2,3_

The inverse Fourier transform analysis for PDW state in Fig. 4c is implemented using the same technique described here in Methods. The filter size chosen to visualize the PDW is 11.4 Å. The inverse Fourier transform of the CDW in Fig. 4d is calculated using an identical filter size of 11.4 Å.

#### Independent PDW visualization experiments

**Extended Data Fig. 6:**
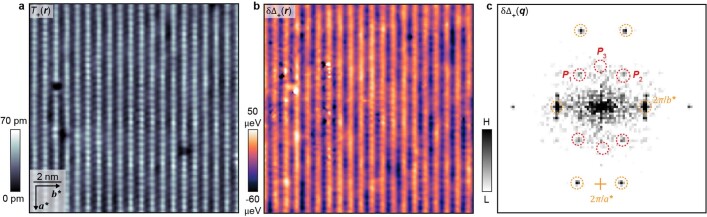
PDW repeatability analysis. **a**, A topograph recorded in a new FOV away from that seen in Fig. 3c. The image size is 15 nm × 15 nm (*V*_s_ = 3 mV, *I*_s_ *=* 2.5 nA). **b**, *δ*Δ_*+*_(**r**) map prepared using the same procedure outlined in Methods shows the same gap modulations as Fig. 4a. **c**, The Fourier transform of *δ*Δ_*+*_(**r**) map, *δ*Δ_*+*_(**q**). (**P**_1_, **P**_2_, **P**_3_) PDW peaks are highlighted with dashed red circles and reciprocal lattice vectors are highlighted with dashed orange circles.

To confirm that the PDW discovered is present in several FOVs, we show a typical example of the gap modulation Δ_+_(**r**) from one different FOV in Extended Data Fig. 6. The ${\rm{d}}I\,/\,{\rm{d}}V{| }_{{\rm{SIS}}}({\bf{r}},V)$ map is measured in the voltage region surrounding the positive Nb-UTe_2_ energy maxima near 1.6 meV. The spectra in this FOV are fitted with a second-order polynomial and shear corrected as described here in Methods. The resulting gap map, *δ*Δ_+_(**r**), is presented in Extended Data Fig. 6b. The Fourier transform of this map, *δ*Δ_*+*_(**q**), is presented in Extended Data Fig. 6c. *δ*Δ_*+*_(**q**) features the same PDW wavevectors (**P**_1_, **P**_2_, **P**_3_) reported in the main text.

### Energy modulations of subgap Andreev resonances

**Extended Data Fig. 7:**
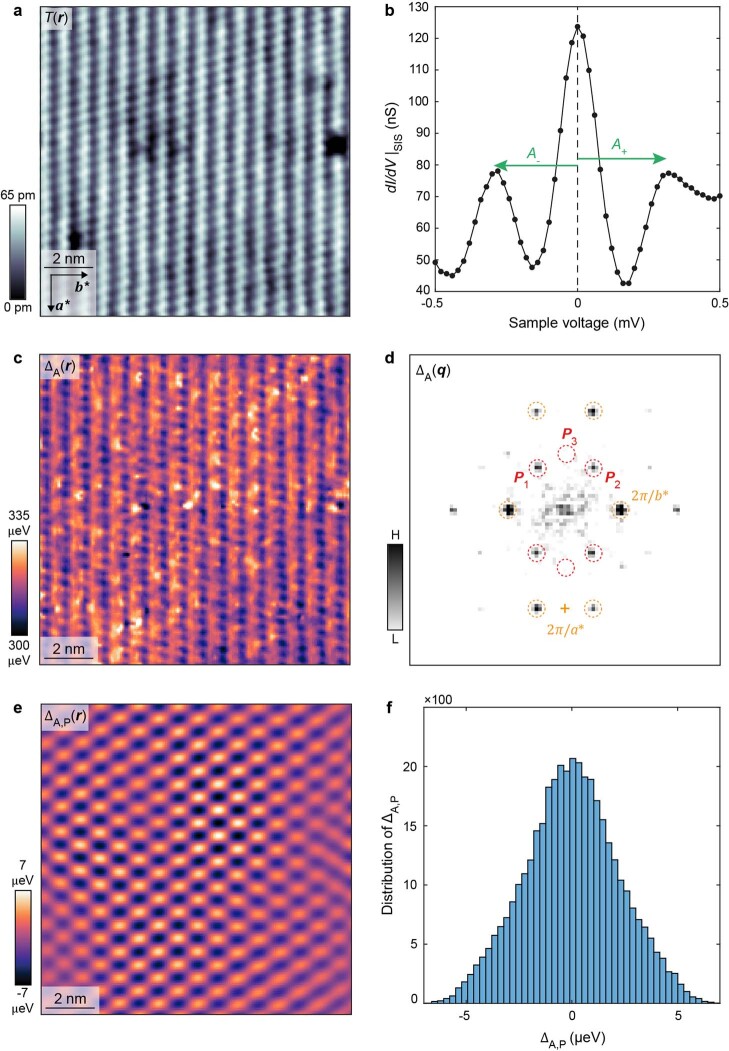
Imaging of subgap Andreev resonances. **a**, Topography of the subgap states imaging in the same FOV as Figs. 3 and 4. **b**, A representative ${\rm{d}}I\,/\,{\rm{d}}V{| }_{{\rm{SIS}}}\left(V\right)$ spectrum of the subgap states annotated by the green arrows. **c**, Map of the energy scale of the subgap states modulations Δ_A_(**r**). **d**, Fourier transform of the subgap states modulations Δ_A_(**q**). **P**_1,2,3_ PDW peaks are highlighted with dashed red circles. **e**, Inverse Fourier transform Δ_A,P_(**r**) of PDW peaks **P**_1,2,3_. **f**, Histogram of Δ_A,P_(**r**) shows that the PDW modulates within 10 µeV.

Surface Andreev bound states must occur in *p*-wave topological superconductors^41^. Moreover, based on the phase-changing quasiparticle reflections at the *p*-wave surface, finite-energy Andreev resonances should also occur in the junction between a *p*-wave and an *s*-wave superconductor^42^ and are observed in UTe_2_. Inside the SIS gap, we measure the ${\rm{d}}I\,/\,{\rm{d}}V{| }_{{\rm{SIS}}}\left({\bf{r}},V\right)$ map in the energy range from −500 µeV to 500 µeV. The map is measured in the FOV in Extended Data Fig. 7a, the same FOV as in Figs. 3 and 4. Three conductance peaks are resolved at approximately −300 µeV, 0 and 300 µeV, annotated with green arrows in the typical subgap spectrum in Extended Data Fig. 7b. The energy maximum of the positive subgap states between 200 µeV to 440 µeV is assigned as *A*_+_. The energy maximum of the negative subgap states between −440 µeV to −200 µeV is assigned as *A*_−_. The averaged energy of the Andreev subgap states is defined as ${\Delta }_{{\rm{A}}}({\bf{r}})\equiv \left[{A}_{+}({\bf{r}})-{A}_{-}({\bf{r}})\right]/2$, which ranges from 300 µeV to 335 µeV (Extended Data Fig. 7c). Fourier transform of the subgap energies Δ_A_(**q**) exhibit two sharp peaks at the PDW wavevectors **P**_1_ and **P**_2_ (Extended Data Fig. 7d).

In the case of two superconductors with very different gap magnitude, when the sample bias voltage shifts the smaller gap edge to the chemical potential, the Andreev process of electron (hole) transmission and hole (electron) reflectional plus electron-pair propagation may produce an energy maximum in d*I*/d*V*|_SIS_ at the voltage of smaller gap energy. Hence, the observations in Extended Data Fig. 7d may be expected if the UTe_2_ superconducting energy gap is modulating at the wavevectors **P**_1_ and **P**_2_. Extended Data Fig. 7e shows that the energy of the Andreev states modulate in space with a peak-to-peak amplitude near 10 µeV (see histogram in Extended Data Fig. 7f).

### Enhancement of signal-to-noise ratio using superconductive tips

Superconducting STM tips provide an effective energy resolution beyond the Fermi–Dirac limit. They have therefore been widely used as a method of enhancing the energy resolution of STM spectra^26–31^.

**Extended Data Fig. 8:**
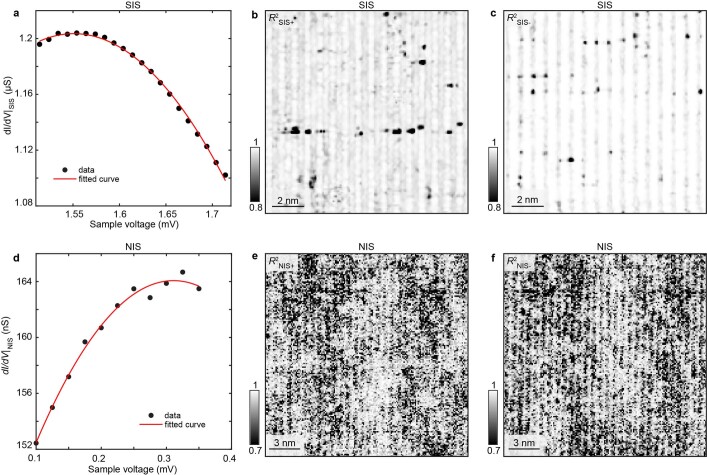
Estimation of signal-to-noise ratio using fitting quality of spectra measured with superconductive tips and normal tips. **a**, Parabolic fit of a typical ${\rm{d}}I\,/\,{\rm{d}}V{| }_{{\rm{SIS}}}$ spectrum measured using superconductive tips. **b**,**c**, Measured *R*^2^ maps used to estimate the fitting quality of ${\rm{d}}I\,/\,{\rm{d}}V{| }_{{\rm{SIS}}}$ spectra for positive energy (**b**) and negative energy (**c**). The *R*^2^ image is from the FOV of Fig. 3c. **d**, Parabolic fit of a typical ${\rm{d}}I\,/\,{\rm{d}}V{| }_{{\rm{NIS}}}$ spectrum taken using normal tips. **e**,**f**, Measured *R*^2^ maps used to estimate the fitting quality of ${\rm{d}}I\,/\,{\rm{d}}V{| }_{{\rm{NIS}}}$ spectra for positive energy (**e**) and negative energy (**f**). The *R*^2^ image is from the FOV in Extended Data Fig. 3b.

To better quantify the signal-to-noise ratio improvement of the measured energy-gap modulations, we compare the fitting quality of the superconducting gap maps obtained using a normal tip (Extended Data Fig. 3) and a superconducting tip (Fig. 4). The fitting quality is defined using the coefficient

22 $${R}^{2}\left({\bf{r}}\right)=1-\frac{{\sum }_{i=1}^{N}{\left[g\left({\bf{r}},{V}_{i}\right)-{\rm{d}}I/{\rm{d}}V\left({\bf{r}},{V}_{i}\right)\right]}^{2}}{{\sum }_{i=1}^{N}{\left[g\left({\bf{r}},{V}_{i}\right)-\bar{g}\left({\bf{r}}\right)\right]}^{2}}$$

in which ${\rm{d}}I\,/\,{\rm{d}}V\left(V\right)$ is the measured spectrum, *g*(**r**, *V*) is the fitted spectrum and $\bar{g}\left({\bf{r}}\right)$ is the averaged fitted spectrum. Extended Data Fig. 8a shows a typical spectrum measured using a superconductive tip, ${\rm{d}}I\,/\,{\rm{d}}V{| }_{{\rm{SIS}}}$ from the FOV in Fig. 3c. Extended Data Fig. 8d is a typical ${\rm{d}}I\,/\,{\rm{d}}V{| }_{{\rm{NIS}}}$ spectrum measured using a normal tip from the FOV in Extended Data Fig. 3. The energy-maximum noise level is decisively lower in ${\rm{d}}I\,/\,{\rm{d}}V{| }_{{\rm{SIS}}}$ spectra than in ${\rm{d}}I\,/\,{\rm{d}}V{| }_{{\rm{NIS}}}$ spectra and the fitting quality ${R}_{{\rm{SIS}}}^{2}$ is substantially higher than ${R}_{{\rm{NIS}}}^{2}$.

Extended Data Fig. 8b,c shows maps of the fitting parameter *R*^2^ calculated from fitting the d*I*/d*V*|_SIS_ energy-maxima map obtained using a superconductive tip, that is, the ${\Delta }_{{{\rm{UTe}}}_{{\rm{2}}}}({\bf{r}})$ images presented in Fig. 3e,f. Extended Data Fig. 8e,f shows maps of *R*^2^ calculated from the coherence peak fitting of d*I*/d*V|*_NIS_ obtained using a normal tip, that is, the ${\Delta }_{{{\rm{UTe}}}_{{\rm{2}}}}({\bf{r}})$ images presented in Extended Data Fig. 3e. Comparing these *R*^2^ quality-of-fit parameter maps, we find that a much larger fraction of normal-tip coherence peak maps have poor correspondence with the fitting procedures used. For superconducting tips, the root-mean-square values of the fitting parameter, ${R}_{{\rm{RMS}}}^{2}$, are 0.98 and 0.99 for the positive and negative coherence peak fitting, respectively. The normal-tip ${R}_{{\rm{RMS}}}^{2}$ values are 0.87 and 0.86 for the positive and negative coherence peak fitting, respectively. The superconducting tip therefore demonstrably achieves a marked signal-to-noise ratio enhancement for evaluation of ${\Delta }_{{{\rm{UTe}}}_{{\rm{2}}}}\left({\bf{r}}\right)$ images.

As the signal-to-noise ratio is increased in the SIS-convoluted coherence peaks measured using a superconducting tip, it has been possible to resolve the UTe_2_ energy-gap modulations of order approximately 10 μV. Fundamentally, the energy resolution is associated with the ability of the superconductive tip to resolve the energy at which the d*I*/d*V*|_SIS_ coherence peak reaches its maximum amplitude. Consequently, we determine our energy resolution to be 10 μV.

Thus, the same superconductor energy-gap modulations in ${\Delta }_{{{\rm{UTe}}}_{{\rm{2}}}}\left({\bf{r}}\right)$ of UTe_2_ can be observed using either a superconducting tip or a normal tip. However, the former substantially increases the SIS conductance at $\left|E\right|={\Delta }_{{{\rm{UTe}}}_{{\rm{2}}}}+{\Delta }_{{\rm{tip}}}$ and allows for considerably better imaging of these energy maxima and thus ${\Delta }_{{{\rm{UTe}}}_{{\rm{2}}}}\left({\bf{r}}\right)$.

### Interplay of subgap quasiparticles and PDW

**Extended Data Fig. 9:**
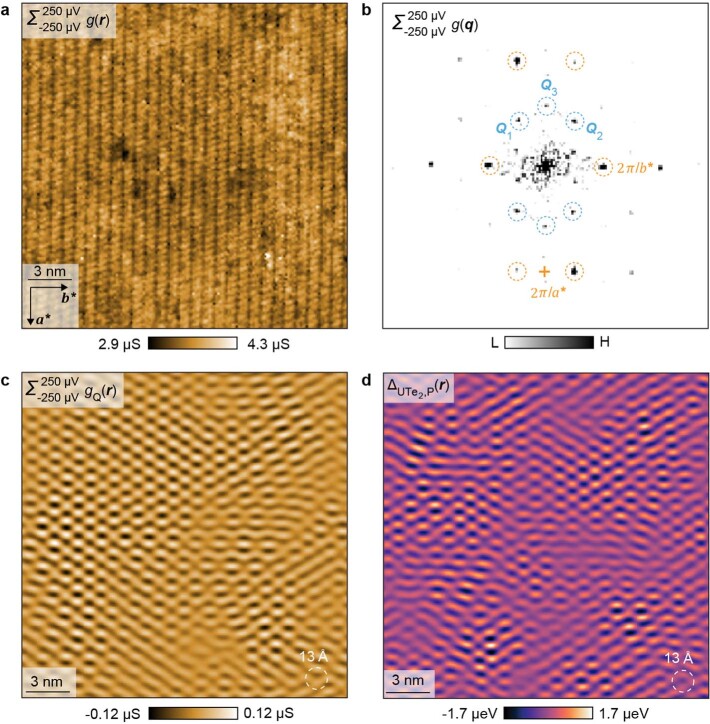
Modulations of subgap states measured using normal tips. **a**, Sum of all subgap states ${\sum }_{-250\,{\rm{\mu }}{\rm{V}}}^{250\,{\rm{\mu }}{\rm{V}}}g\left({\bf{r}},E\right)$, measured at *T* = 280 mK. **b**, Fourier transform of subgap states ${\sum }_{-250\,{\rm{\mu }}{\rm{V}}}^{250\,{\rm{\mu }}{\rm{V}}}g\left({\bf{q}},E\right)$, in which all three wavevectors **P**_1,2,3_ are present. **c**, Inverse Fourier transform of **P**_1,2,3_ from **a**. **d**, Inverse Fourier transform of **P**_1,2,3_ from Extended Data Fig. 3e,f. The filter size is indicated as a dashed white circle.

Here we show simultaneous normal-tip-measured modulations of the UTe_2_ subgap states and ${\Delta }_{{{\rm{UTe}}}_{{\rm{2}}}}\left({\bf{r}}\right)$ at *T* = 280 mK, to study their interplay. Extended Data Fig. 9a shows the integrated differential conductance from −250 μV to 250 μV, ${\sum }_{-250\,{\rm{\mu }}{\rm{V}}}^{250\,{\rm{\mu }}{\rm{V}}}g\left({\bf{r}},E\right)$. Inverse Fourier transform of the three wavevectors **Q**_1,2,3_ from ${\sum }_{-250\,{\rm{\mu }}{\rm{V}}}^{250\,{\rm{\mu }}{\rm{V}}}g\left({\bf{r}},E\right)$ and **P**_1,2,3_ from the simultaneous ${\Delta }_{{{\rm{UTe}}}_{{\rm{2}}}}\left({\bf{r}}\right)$ in Extended Data Fig. 3e are compared in Extended Data Fig. 9c,d. Clearly, from the highly distinct spatial structure of these images, there is no one-to-one correspondence between the subgap density-of-states modulations and the simultaneously measured PDW energy-gap modulations in UTe_2_. Overall, there is a very weak anticorrelation, with a cross-correlation value of −0.23 ± 0.05 that is not inconsistent with coincidence. Hence we demonstrate that there is no deterministic influence of the subgap density-of-states modulations on the PDW energy-gap modulations in superconducting UTe_2_.

### Visualizing the interplay of PDW and CDW in UTe_2_

**Extended Data Fig. 10:**
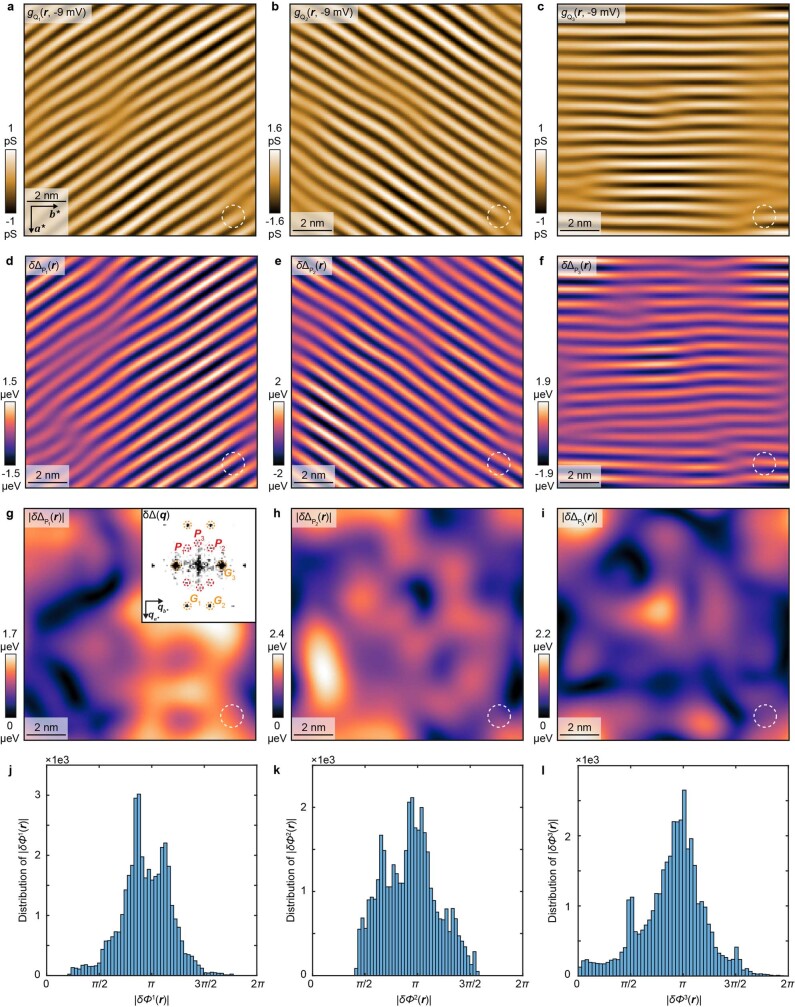
Phase shift between CDW and PDW. **a**–**c**, Inverse Fourier transforms of the three CDW wavevectors identified ${g}_{{{\bf{Q}}}_{i=1,2,3}}({\bf{r}},-\,9\,{\rm{mV}})$ in the same 12 nm × 12 nm FOV as Fig. 3c. **d**–**f**, Inverse Fourier transforms of the three PDW $\delta {\Delta }_{{{\bf{P}}}_{i=1,2,3}}$ wavevectors in the same FOV as Fig. 3c. **g**–**i**, Amplitude for all three PDW wavevectors **P**_*i*=1,2,3_. Inset of **g** is the Fourier transform of the energy gap map, in which the reciprocal lattice points **G**_*i*=1,2,3_ are labelled. **j**–**l**, Distributions of the relative spatial phase difference $\delta {\phi }_{i}({\bf{r}})$ between ${\phi }_{i}^{{\rm{C}}}\left({\bf{r}}\right)$ and ${\phi }_{i}^{{\rm{P}}}\left({\bf{r}}\right)$ from three individual wavevectors. Each histogram is centred around π, reinforcing the observation of a general phase difference $| \delta {\phi }_{i}| \cong \pi$ between the CDW and the PDW.

The analysis of phase difference between PDW and CDW at three different wavevectors is shown in Extended Data Fig. 10. The inverse Fourier transforms of each CDW and PDW wavevector demonstrate a clear half-period shift between the two density waves (Extended Data Fig. 10a–f). This shift motivates the statistical analysis of the phase difference. The phase map of ${g}_{{{\rm{Q}}}_{1}}({\bf{r}},-9\,{\rm{mV}})$, ${\phi }_{1}^{{\rm{C}}}\left({\bf{r}}\right)$, and the phase map of ${\Delta }_{{{\rm{P}}}_{1}}({\bf{r}})$, ${\phi }_{1}^{{\rm{P}}}\left({\bf{r}}\right)$, are calculated. The phase difference between two corresponding maps is defined as $| \delta {\phi }_{1}| ={\phi }_{1}^{{\rm{C}}}\left({\bf{r}}\right){\boldsymbol{-}}{\phi }_{1}^{{\rm{P}}}\left({\bf{r}}\right)$ for the **P**_1_:**Q**_1_ wavevectors. Identical procedures are carried out for **P**_2_:**Q**_2_ and **P**_3_:**Q**_3_. The histograms resulting from this procedure show that the statistical distributions of the phase shift $|\delta {\phi }_{i}{\rm{|}}$ are centred around π (Extended Data Fig. 10j–l). Although the distribution varies, this π phase shift reinforces the observation of the spatial anticorrelation between CDW and PDW.

As shown in the inset of Extended Data Fig. 10g, the three PDW wavevectors are related by reciprocal lattice vectors: **P**_2_ = **P**_1_ − **G**_3_ and **P**_3_ = **G**_1_ − **P**_1_. Nevertheless, the three UTe_2_ PDWs seem to be independent states when analysed in terms of the spatial modulations of the amplitude of the **P**_1,2,3_ peaks from Fig. 4 using equation (16). The amplitude of **P**_1,2_ has a domain width beyond 10 nm in the real space (Extended Data Fig. 10g,h). The amplitude of **P**_3_ is short-ranged, of which the averaged domain width is approximately 5 nm (Extended Data Fig. 10i). The one-pixel shift of **P**_3_ from the central axis is within the error bar of experimental measurements. The spatial distributions of the three PDWs are negligibly correlated with cross-correlation values of their amplitude of *X*(**P**_1_, **P**_2_) = −0.3, *X*(**P**_1_, **P**_3_) = 0.9 and *X*(**P**_2_, **P**_3_) = 0.28. The weak cross-correlation relationships indicate that the three PDWs are independent orders.

## Online content

Any methods, additional references, Nature Portfolio reporting summaries, source data, extended data, supplementary information, acknowledgements, peer review information; details of author contributions and competing interests; and statements of data and code availability are available at 10.1038/s41586-023-05919-7.

## Data Availability

The data shown in the main figures are available from Zenodo at 10.5281/zenodo.7662516.

## Code availability

The code is available to qualified researchers from the corresponding authors on reasonable request.
